# Renewable Energy from Beach-Cast Seaweed: Calorific Power Heating Studies with Macroalgae

**DOI:** 10.3390/plants14071005

**Published:** 2025-03-23

**Authors:** Fernando Pinto Coelho, Everardo Valadares de Sá Barreto Sampaio, Márcio Gomes Barboza, Elica Amara Cecília Guedes-Coelho, Manoel Messias da Silva Costa, Emerson Carlos Soares da Silva, Victor Andrei Rodrigues Carneiro, Bruno Moreira Soares, Elvis Joacir de França, Rômulo Simões Cezar Menezes, Cesar Augusto Moraes de Abreu

**Affiliations:** 1 Postgraduate Program in Energy and Nuclear Technologies, Federal University of Pernambuco (UFPE/PROTEN), National Nuclear Energy Commission, Recife 50740-540, Brazilrmenezes@ufpe.br (R.S.C.M.); moraedeasbreu@gmail.com (C.A.M.d.A.); 2 Research Nucleus in Energy Production, National Council for Scientific Research CNPQ, Brasília 70070-010, Brazil; 3 Surveying and Cartography Engineering Course, Center of Engineering and Agricultural Sciences, Federal University of Alagoas, Campus—(CECA), Rio Largo 57072-016, Brazil; 4 Civil Engineering Technology Centre, Federal University of Alagoas, Campus A. C. Simões, Maceió 57000-000, Brazil; mbarboza@ctec.ufal.br; 5 Institute of Biological and Health Sciences (ICBS), Federal University of Alagoas, Campus A. C. Simões, Maceió 57000-000, Brazil; eac.guedes@gmail.com; 6 Federal Institute of Alagoas/IFAL, Brazilian Regional University (UNIRB), Maceió 57035-660, Brazil; manobio@hotmail.com; 7 Aquaculture and Aquatic Ecology, Laboratory of Center of Engineering and Agricultural Science, Federal University of Alagoas, Campus—(CECA), Rio Largo 57072-016, Brazil; soaemerson@gmail.com; 8 Institute of Biological Sciences, University of Sao Paulo, São Paulo 05508-220, Brazil; victorandreirc91@gmail.com; 9 Institute of Science, Technology and Innovation—Proalga Brazil—ICTI, São Paulo 01109-060, Brazil; bruno.soares@proalgabrasil.org.br; 10 Northeast Regional Centre for Nuclear Sciences, Recife 50740-545, Brazil; ejfranca@gmail.com

**Keywords:** biomass, bioenergy, calorific value, combustion, macroalgae, pellets

## Abstract

Some stretches of the Brazilian coast are regularly subject to a natural process of macroalgae deposition. In urban beach areas, public institutions responsible for cleaning collect this biomass and dispose of it in landfills. When this biomass is exposed to the sun for a long time in the littoral area, a decomposition process begins and causes greenhouse gas emission into the atmosphere. Macroalgae biomass is a natural resource that could be used for renewable energy, contributing to meeting the growing demand for low environmental impacts of energy, indicating the possibility of participating in sustainable development. The objective of this research was to evaluate the energetic potential of macroalgae biomass deposited on the Maceió coast; specifically, the combustion capacity of aggregate biomass and pellet biofuel produced with macroalgae. The research, which analysed 13 species, proceeded using a calorimetric pump methodology to determine the power heating value and a mass spectrophotometer to determine the available energetic chemical elements. The result of 8.82 MJ/Kg was similar to the main biomass used in Brazil, the sugarcane bagasse, evaluated at 8.91 MJ/Kg. Aggregated macroalgae biomass in condensed pellets with energetic composites obtained a value of 4823 Kcal/Kg, 1.2% more than the average of terrestrial biomass pellets. Therefore, these results show possibilities to produce biofuel using thermal energy from marine macroalgae biomass.

## 1. Introduction

The macroalgae market is centred on the production, processing, and distribution of macroalgae, which is more commonly referred to as seaweed. This industry exerts a multifaceted influence on various sectors, including food and beverages, pharmaceuticals, cosmetics, and biofuels. The growing recognition of macroalgae as a nutritious food is due to their high content of vitamins, minerals, and antioxidants. This heightened awareness has led to a surge in their incorporation into health foods and dietary supplements, aligning with the preferences of health-conscious consumers [1].

The industrial production systems associated with energy production actively use natural resources and act as consumers of the main raw materials found in nature. These intensified practices claim concerns about environmental defence preservation, life protection and sustainable development [2,3,4,5]. Energy consumption has increased by 65% in the last 30 years and will increase by another 40% by 2030, with investments reaching $600 billion per year [2]. The investment in renewable energy exceeded USD 600 billion per year in 2020 [6,7,8]. Renewable energies offer a viable alternative for the future provision of energy, with a view to the enhancement of biofuel production and the improvement of quality of life for citizens, who will no longer be subject to the constraints imposed by their reliance on fossil fuels. [4]. The algae are thallophyte plants lacking roots, stems, and leaves, with chlorophyll “a” as their primary photosynthetic pigment and they need a sterile covering of cells around the reproduction system [9]. The marine ecosystem is composed of phytobenthic macroalgae [10,11,12], and some species may present an oleaginous concentration composed of fatty acids, with the potential to generate heat in thermoelectric and biofuel industries [13,14,15]. Marine macroalgae biomass is renewable in continental ocean ecosystems and can be an important raw material for new biofuel energy generation. The efficient enhanced growth photosynthesis, which is four times higher than terrestrial plants, became an important factor concerning sustainability [16,17,18,19]. The beach-cast seaweed enables a free daily biomass deposition in various littoral parts of the world; it is the only kind of biomass collected every day and the raw material can be used for energy production, dispensing with irrigation use, pesticides, fertilizers and agricultural inputs. The findings of this study contribute to the existing body of knowledge concerning the utilization of residual biomass for energy generation purposes. In Maceió, the municipal collection service employs a daily removal process that utilizes three specialized buckets with a capacity of six tons of macroalgae aggregated biomass with residual sand. This amount has been invariably discarded in the sanitary landfill and characterized as unusable. The use of macroalgae biomass opens up possibilities for the production of biofuels with new technologies and emphasizes the creation of another sustainable alternative to help overcome the current global energy crisis and reduce its environmental problems. In the research, the combustion capacity of aggregated biomass and pellets was evaluated in relation to the calorific value obtained. It is important to highlight this research in Brazil as the first that produced seaweed pellets with a focus on the energy potential of biomass.

## 2. Results and Discussion

### 2.1. The Thermal Capacity of Macroalgae from HCV and LCV

Some species in this research presented higher calorific value (HCV) than others, ranging from 6.3–12.0 MJ/Kg, and lower calorific value (LCV) 5.9–10.8 MJ/Kg—(Table 1), which these values are under than most terrestrial biomasses in according [17,20]. The lower calorific value—8.82 MJ/Kg, (Table 1), was similar to the main biomass used in Brazil, sugarcane bagasse, evaluated at 8.91 MJ/Kg [21]. This result configures an economic possibility to use this marine biomass. Among the factors that could explain the inferior position of HCV of macroalgae in relation to terrestrial biomass, we can mention nearing absence of lignin, high humidity and less concentrations of carbon and hydrogen than terrestrial biomass [22,23]. (Table 2). A selective formulation composed of 25% *Sargassum* sp., 35% *Cryptonemia crenulata*, 10% *Gracilaria* sp., 30% *Sargassum vulgare*, presented an estimated higher calorific value of 11.29 MJ/Kg, about 11.2% higher than aggregated biomass formulated with all macroalgae species—10.09 MJ/Kg (Table 2), meaning the possibility for selective collection of species with better yield and greater energy potential. According [24], the phylum Chlorophyta and Ochrophyta have higher calorific values, respectively, 8–13 MJ/Kg–9–11 MJ/Kg, indeed, the average found in the present research in both phyla are 8.67MJ/Kg and 10.19 MJ/Kg, which are almost similar range cited in the literature (Table 1). The species of phylum Rhodophyta reached the highest calorific value, 11.4–12.0 MJ/Kg (Table 1). For [25,26], the HCV values of macroalgae are in the range of 11–12 MJ/Kg, lower than those of terrestrial biomasses, which are situated in the range of 17–18 MJ/Kg. Research by [27,28] with macroalgae provided HCV of 17.6 MJ/Kg and 21.7 MJ/Kg, respectively. Along the same high calorific levels, but with other species and from different methods, through performed pyrolysis with macroalgae *Laminaria japonica*, *Fucus serratus* and *Prophyra tenera* at 500 °C, have reached results of 33.57 MJ/Kg; 32.46 MJ/Kg and 29.74 MJ/Kg, respectively [29]. These results are superior to those many terrestrial biomasses. Thus, indications of macroalgae biodiversity configure different chemical compositions in their elements, some aspects of seasonality, environment, regionality and differentiated genetic evolution than terrestrial plants, may, in certain cases, show higher potential energy when compared of terrestrial biomass [29,30,31,32].

#### 2.1.1. Chemical Combustion Elements for Energy Production—Analysis of C, H, N, P, K, O

Research by [33,34], indicated that raw materials with a high content of carbon and hydrogen have a high calorific value, while the presence of oxygen has the opposite effect. The species *Ulva lactuca* and *Hypnea pseudomusciformis* contain high levels of carbon mass—50.08% and 61.87% with HCV levels as 11.43 MJ/Kg and 8.73 MJ/Kg respectively, (Table 1 and Table 2), while can be compared with result obtained by [24,35] for the specie *Ulva lactuca*, of 12.89 MJ/Kg, favouring the use of macroalgae carbon species composition for bioenergy, with possible specific offshore crops marine farms [36].

According [37], the high carbon content of some macroalgae species may be due they have several sulphated polysaccharides (PSs) in their composition, characterizing *Hypnea pseudomusciformis* specie as Rhodophyta carrageenan, a family of PSs that undergo variations originating from free hydroxyl substitutions. These polymers are formed by the repetition of disaccharide units.

#### 2.1.2. Analysis of Combustion Macroalgae Biomass to Get Bioenergy

Combustion efficiency assessments are based on the results of biomass calorific value. Macroalgae have an average moisture content over 70%, which could be a factor that prevents efficient combustion of their biomass, reducing its calorific value. Although this moisture content varies with the species, extraction processes and technological drying methodologies can make all the difference for efficient combustion [38]. Energy conversion losses are shown to be effective between the lower calorific value and the higher calorific value, this difference from 8.82 MJ/Kg to 10.09 MJ/Kg and 20.19 MJ/Kg for pellets, characterizes the combustion efficiency for raw materials condensed into energy composites, synthesizing greater sustainability for briquettes and pellets produced. The main gases emitted during macroalgae combustion are carbon dioxide (CO_2_) and water vapor (H_2_O).

These are the typical products of biomass combustion. CO_2_ and H_2_O are formed when the organic compounds present in macroalgae, such as carbohydrates and lipids, react with oxygen during the burning process. It is important to note that although macroalgae emit CO_2_ during combustion, they also absorb CO_2_ during their growth through photosynthesis [39,40]. This creates a shorter carbon cycle compared to fossil fuels, potentially reducing the net impact on greenhouse gas emissions [40]. Mitigation process with macroalgae cultivation can act as a carbon sink, absorbing between 0.46–2.55 Pg. of carbon per year [41]. In addition, depending on the specific composition of the macroalgae and combustion conditions, other gases can be emitted in smaller quantities, such as nitrogen oxides—NOx (1–5%) and sulphur compounds (1–2.5%), due to the presence of these elements in the algae biomass [34,40]. Macroalgae produce ash with a high alkaline metal content, which can cause fouling and corrosion in combustion systems.

The co-firing of macroalgae with other fuels has several potential benefits [39,41]: (1) Reduction of emissions: Co-firing can reduce total emissions per unit of energy produced compared to burning fossil fuels alone. (2) Reduction of pollutants: There is potential to reduce emission levels of pollutants such as sulphur oxides, heavy metals, dioxins and furans, due to the composition of macroalgae. (3) Use of waste: The use of macroalgae deposited on beaches, which would otherwise be discarded, minimises waste and reduces transport and disposal costs. (4) Renewable resource: Macroalgae are a source of renewable biomass, contributing to the diversification of the energy matrix. (5) Short-term carbon cycle: Although they emit CO_2_ during combustion, macroalgae absorb CO_2_ during their growth, potentially reducing the net impact on greenhouse gas emissions [34]. (6) Energy potential: Some species of macroalgae have a calorific value comparable to that of terrestrial biomass used for energy generation. (7) Mitigation of environmental impacts: Co-firing can reduce soil and water pollution, depending on the chemical composition of the material used [34,39]. It is important to note that, despite these potential benefits, macroalgae co-firing still faces technical and economic challenges that need to be overcome for its large-scale implementation. Direct combustion of macroalgae presents significant challenges due to their unique chemical composition, but their potential as a renewable energy source and carbon sink merits further research and technological development [40].

Biofuels produced with macroalgae have already demonstrated efficiency in the production of ethanol for automotive vehicles, achieve a title of 4.7% volume/volume and a yield of 0.281 weight ethanol/weight dry macroalgae [33], equivalent around 80% of the maximum theoretical yield from the sugar composition in macroalgae [42] and still by pellets, can also be adapted more sustainably for use in bakery ovens, pizzerias and industrial boilers that normally use non-certified wood or biomass with low calorific value.

### 2.2. Meaning Relation About Fibre Elements Energy in Macroalgae

The lignin rates in this research ranged from 0.31% to 13.74% are low (Table 3). The studies and analysis of [10], demonstrated that macroalgae lignin and cellulose have a reduced calorific value compared to terrestrial biomass, it means that lignin HCV has an average of 0.025 MJ/Kg against 0.015 MJ/Kg for celluloses. It is known that the lignin polymer contains less oxygen than the polysaccharides present in holocellulose [43]. This factor distinguishes in terms of its higher HCV value. The cellulose content between 5.13–23.29%, around 11.28% average (Table 4), it’s not so different by research of [44], when the different chain molecules of cellulose glucose polymer is present in Ochrophyta, Chlorophyta and Rhodophyta with 10%. Reduced cellulose modified in macroalgae, compared to terrestrial plants, would can be caused by the fact that phenolic compounds present in the cell walls of macroalgae are interacting as inhibitors in several development processes; as about cellular level, by lipid metabolism influences, and his biochemical mechanism of respiration, inhibiting glucose transport and cellulose synthesis, it means, contains in quantity the compound responsible for hydrolysis that decomposes the cellulosic polymer [43]. A fibrillar structure is important for the physical reinforcement of cell walls. However, other researches like [44,45], reported that some species, such as *Gracilaria verrucosa*, reach 60% cellulose, and [46], for a kind of *Vallonia* specie with more than 70% cellulose. According to [47], cellulose levels in macroalgae depend on different biomass treatment systems, Methane yields, have been improved by 19–68% after the breakdown of biomass structures by mechanical, thermal, enzymatic, and chemical treatments to improve cellular access to polysaccharide-hydrolysing agents [48,49]. Cellular variability in macroalgae membranes can determine the beneficial value of mechanical treatment, where those with more fibrous cell walls would benefit the decrease in its size [50]. Macroalgae exhibit distinct characteristics in the cell wall [51] standing out these differences as brown algae present cellulose in the primary skeletal cell wall, while green and red algae present xylose, mannose and cellulose [52]. This context of low fibre composition can be attributed to the absence of roots, stems and leaves in macroalgae [53]. This factor indicates better possibilities to biogas generation than terrestrial plants, when you have applicate anaerobic digestion and hydrolysis processes, due to the greater ease of permeation inside the plant, being free of lignin barrier in organic matter degradation processes.

According [17,27,29]; The macroalgae ash content is higher than in terrestrial plants. Some species varying 18–55% [54,55]. Therefore, following the analysis of 10 species, the author yielded results ranging from 14–39.7% to 3.3–46% in four species examined [56]. The results in the present research with 11 species analysed found 3.75% for aggregated biomass. It cannot be said that species with the highest ash content, respectively, *Padina* sp. and *Caulerpa microphysa*, (Table 3) achieved high calorific value; which means, this element does not translate efficiency calorific energy to use macroalgae.

These different results can be explained because they are not the same species and indeed, this may a present distinct genetic development [1]. Consonant to [29,30], this variation in ash composition is influenced by seasonality in the appearance of species, with more content ashes at certain times of the year.

This regional instable seasonality was observed in the present research in collect process with the appearance of three new species in the summer season; *Caulerpa escapiliformes*, *Bryopsis plumosa* and *Pictyor mentensii* with predominance of *Bryopsis plumosa* specie from the phylum Chlorophyta. The studies of [57] evaluated that the amino acid content in *Saccharina latissima* in august was almost double in June and ash mineral contents also increased [58]. It is possible to affirm that three species with the lowest content of lignin, cellulose and ash; *Cryptonemia crenulata*, *Gracilaria* sp., *Hydropuntia cornea*, were that presented the highest indices of calorific value. The last two species with less fibre, had elevated potassium levels, establishing a relationship that less fibre with greater calorific value and more potassium content.

### 2.3. Sustainability of Macroalgae Biomass Deposition

**Figure 1:**
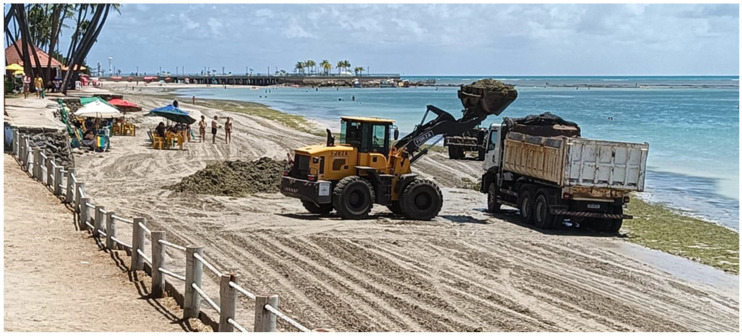
Tractors remove seaweed biomass from Maceió coast beaches (2025).

The sustainability of macroalgae biomass deposited daily along the coast of Alagoas was proven in sampling studies with an average of 5.03 ton/ha [59]. The main reason for this high deposition it is because there is a single harvest per year to terrestrial plants and it is possible to make two collections daily by beach cast seaweed. Environmental problems with marine biomass, such as output greenhouse gases by methane emissions after degradation of organic raw material in high temperature, meaning subjected to long time sun exposure in coastal band area. Therefore, accumulated seaweed biomass transported to landfills every day, would be mitigated with macroalgae biorefinery process. According to the Municipal Secretariat for Sustainable Development of Maceió, two full trucks are stored every day, equivalent to 4 tons of biomass, which is sent to landfills and contributes to high costs for public finances (Figure 1).

### 2.4. Evaluation of Pellets Produced with Heat Capacity

According to [60], the increase abrasion resistance and reduction wear equipment in the production of biofuel from pellets can be achieved with the addition of natural binders such as corn potato starch, sugarcane molasses, vegetable oil and sulfonated lignin (waste from the pulp and paper industry).

**Figure 2:**
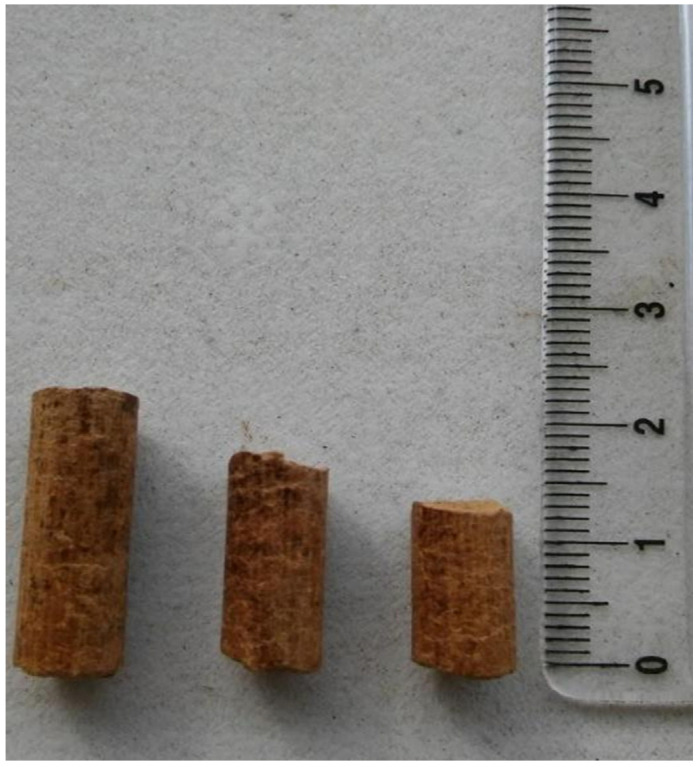
Macroalgae pellets biomass.

**Figure 3:**
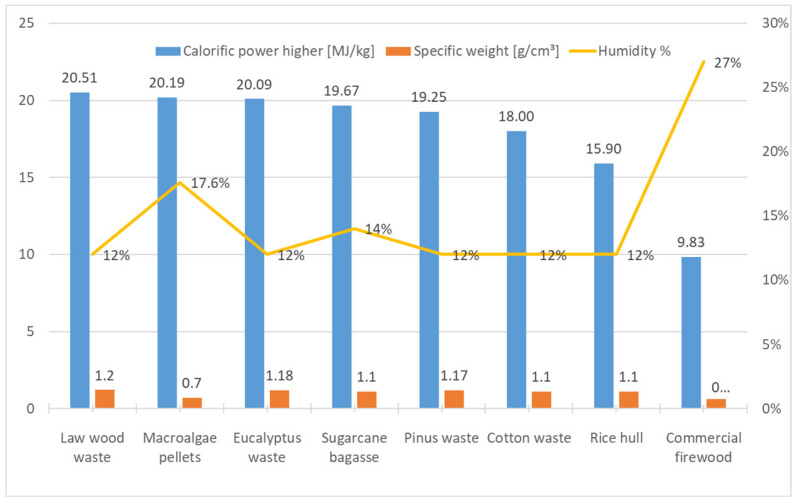
Calorific value comparison of energetic condensates composite from terrestrial biomass against seaweed pellets.

The cylindrical pellets result with granulometry 3 to 5 mm diameter, with a length 8 to 25 mm and a specific mass of 680 Kg/m^3^, was obtained the value of 20.19 MJ/Kg, being produced 1.28 Kg of pellets with 6 Kg of natural biomass. (Figure 2 and Figure 3). It follows, the corn oil binder participated in the HCV with a contribution of 1.88 MJ/Kg, representing 9.35% of calorific value. In the present work there was a loss of calorific energy by content biomass moisture of 17.61% in the pellets.

According [61] reported that additive uses should be analysed with caution because sulfonated lignin, for example, increases the sulphur content causing undesirable gas emissions when combustion of pellets is evaluated for environmental impact. There is no consensus on the use of binders, in the United States and Italy the use of these additives is limited for some products, to get stamp high quality pellets is not allowed. In Sweden, the use of these binders must be indicated on the product packaging.

Research conducted by [62] in Sweden showed that producers in this country use 0.5 to 2.0% potato starch in wood pellets. A comparison of the higher calorific values of terrestrial biomasses condensed into energetic composites of briquettes versus macroalgae aggregated into pellets (Figure 2) shows 9.83–20.51 MJ/Kg, with an average of 17.61 MJ/Kg, which is 11.46% lower than the higher calorific value of energetically condensed pellets—20.19 MJ/Kg (Figure 3). The higher calorific value of different pellets studied by [63] was also inferior to macroalgae pellets (Table 4). The calorific values of pellets and briquettes are equivalent because they are compacted in the same structure and show no significant differences when analysed for the same biomass, since the compression and moisture suppression processes are similar in the two cylindrical formats.

Another lower calorific value study by [64], with 17 species of pellets, obtained an average LCV of 16.10 MJ/Kg, were also 11.6% lower than macroalgae pellets with LCV of 18.76 MJ/Kg analysed by standard method in this research.

**Table 4:** Higher calorific value of pellets.

Types of Pellets	H.C.V. MJ/KG	References	Authors
Wood of Denmark	20.08	Bruhn et al. (2011)	[58]
Wood of Belgium	20.31	V.K. Verma et al. (2012)	[63]
Finland peat	21.63	V.K. Verma et al. (2012)	[63]
Reed canary grass pellets of Finland	19.25	V.K. Verma et al. (2012)	[63]
Poland apple juice from Industrial waste	20.68	V.K. Verma et al. (2012)	[63]
Pectin from citrus shell—Denmark	19.24	V.K. Verma et al. (2012)	[63]
Sunflower husks—Ukraine	20.27	V.K. Verma et al. (2012)	[63]
Belgium wheat straw	18.25	V.K. Verma et al. (2012)	[63]
Macroalgae—Brazil	20.19	Coelho, F. P. (2024)	[59]

## 3. Material and Methods

### 3.1. Collection Methodology: Description of Macroalgae Sampling Studies

**Figure 4:**
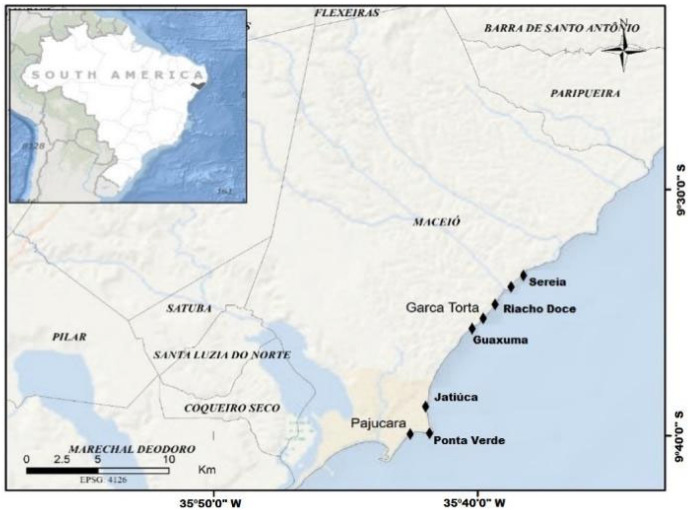
Detailing of macroalgae biomass collection area.

**Figure 5:**
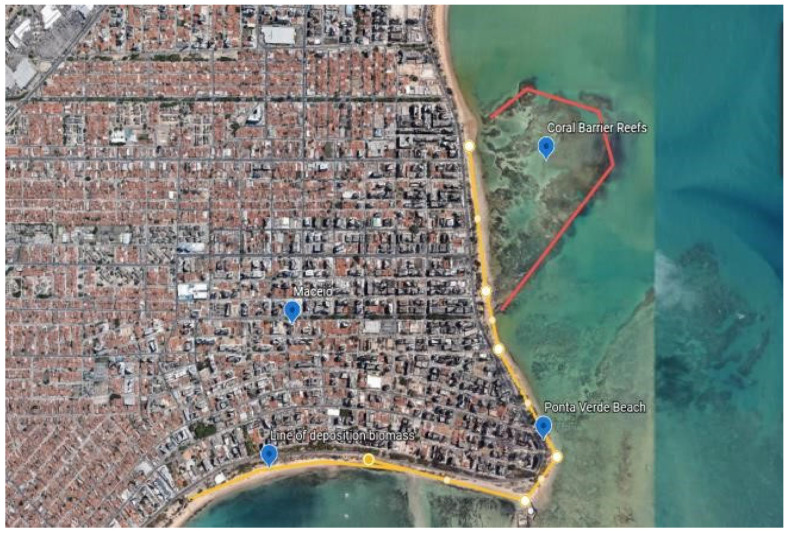
Collection points of macroalgae biomass—Ponta Verde beach in Maceió, Alagoas.

The research area, estimated to be 408.736 m^2^, was georeferenced with the geographic coordinates from the first beach (Pajuçara), given as latitude 9°40′53″ S, longitude 35°42′19″ W, to the last beach (Sereia), with latitude 9°35′17″ S, longitude 35°38′49″ W, as in Figure 4. The sizing of the area was performed with the reference collection points made with the My GPS Coordinates application and images from the NOAA, U.S. Navy, NGA, and GEBECO satellites (Google Maps base, Figure 4 and Figure 5). The analyses samples further collections composed by macroalgae species were carried out from 2014 to 2016, on the beaches of Pajuçara, Ponta Verde, Jatiúca, Guaxuma, Garça Torta, Riacho Doce and Sereia, on the north coast of the municipality of Maceió, Alagoas, Northeast of Brazil.

The algae samples were collected manually, weighed in loco with “EEEKIT” Portable Fishing Scale, with measurement of the gross weight, evaluating the losses with sand, water and garbage founded in biomass. Therefore, washing was carried out in tap water in a stainless-steel tank measuring 55 cm × 48 cm, adopting the decanting method which the heaviest solids, such as sea sand, were deposited at tank bottom and lighter garbage was removed manually from the surface level water. The final residue of fine sand was removed by washing algae in running water superimposed on 50/60 cm aluminium screen sieves. The moisture from biomass was removed by manual pressing to extract the largest volume of water. The collected material was transported to Phycology Laboratory of Federal University of Alagoas, where was identified 27 species taxonomically based on the works described by following authors [65,66,67,68,69], being that 13 species were randomly selected for characterization in a mixture constituted as “aggregated biomass”. The scientific identification names of the species were confirmed through database Algae Base [9]. The drying final process was submitting the biomass to sun exposition during three days in the open air.

#### Collection Methodology by Moon Tide Table

The collection time was determined by the lowest point of tide moon height, when deposition biomass is intense on coast during 2 h and are determined by moon phases, which magnetism exerted by sun and moon forming a strong linear conjunction with our planet, making tide recede from coastal maritime zone with more intensity in sometimes period of the day, alternating in summer waning and full moons, in winter the new and crescent moons, adopting the methodology collection applied by [70]. The height measures of the tides are adopted by Port Authority of the Brazilian Navy and Directorate of Hydrography and Navigation-DHN (Table 5).

### 3.2. Macroalgae Characterization for Evaluation Capacity Energy Calorific Power

The total C and N samples contents were carried out by combustion at 925 °C and determined in a CHNS-O elemental analyser (Perkin Elmer PE-2400, Shelton, CT, USA), using the thermo reference standard (1.755% of C; 0.195% of N and 0.039% S). The samples were dried in oven circulation air at 65 °C for 24 h and passed through 100 mesh sieves. Approximately 3 mg of macroalgae sample was used. Phosphorus and potassium were carried out at Northeast Regional Centre for Nuclear Sciences—CRCN, with an energy dispersive x-ray fluorescence spectrophotometer, EDX 720 equipment, using 10 mm polypropylene collimator to control radiation. The samples were placed in container diameter with 31.6 mm, by volumetric capacity of 10 mL, sealed with polypropylene film. The equivalent mass of each sample was 0.5 to 1 g. The reading time when the sample was irradiated with an X-ray fluorescent tube were detected, from 100 to 300 s, and the respective materials were processed from the standard references SRM 1570a, SRM 1547, SRM 1515. Lignin, Cellulose and Ash quantifications were performed in an ANKON (Macedon, NY, USA) fiber determiner according to described method by [71]. The digesting technique consisted of 0.5 g of seaweed sample in bags made of synthetic material, resistant to digestion, previously weighed and dried in an oven at 65 °C for 24 h.

### 3.3. Analysis of Algae Calorific Value

The high calorific value (HCV) of macroalgae biomass was determined from 13 species with three replicates for each sample, and five replicates for the aggregate biomass sample. The HCV determination was made in an IKA WERKE calorimeter pump (Staufen, Germany), C2000, with 0.6 to 1.0 g raw material placed in a crucible for each sample, into a stainless-steel tank with high pressure (30.0 bar) oxygen atmosphere, which was closed and immersed in a double wall vessel containing 4 L of water.

The water temperature of 23–28 °C was programmed as a function of combustion time relative to biomass amount. For each measurement, with specific heat of the container between 12–28 °C and the water pressure between 1 bar with maximum of 1.5 bar, for energy released during the combustion process was evaluated. The lower calorific value (LCV) of the species and pellets were evaluated using the Standard Method ASTM D-240-64 [72]. The combustion tests based on the HCV results to percentages of hydrogen contained in the biomass by Equation (1):

LCV = HCV − 50.68 × H(1)

It means; LCV is the lower calorific value in calories per gram, HCV is the high calorific value in calories per gram, and H is the percentage of hydrogen in the sample.

### 3.4. Energetic Pellets Condensates

The macroalgae pellets production was carried out after washing and drying biomass in open air. The dry biomass with 6 Kg without milling was placed in pelletizing machine AF-150, 4 Kw, added with 50 mL of corn oil binder, equivalent to 449.5 kcal (1.88 MJ/Kg). The pellets were compacted through by extrusion process in a pellet machine with pressure exerted around 300 MPa or 3059 Kg f/cm^2^, approximate temperature of 120 °C, like similar process by [50]. The superior heat calorific value of aggregated biomass from 13 species in form of pellets was evaluated in a calorimetric bomb, referring based on analysis of five replicates to each one pellet, included three sample. The determination of moisture (M) in the aggregate biomass of pellets was calculated by wet weight (Ww) minus dry weight (Dw) divided by the wet weight × 100. Equation (2)

M = Ww − Dw/Ww × 100(2)

## 4. Conclusions

The aggregated macroalgae biomass can become a new generation of renewable biofuels, reducing the environmental liabilities accumulated in dumps and landfills with elevated costs for public management. Indeed, it’s a free biomass availability with large scale deposition by tropical and continental seas all over the world. Allows efficient use of own energy by achieving a calorific value similar to one of the main biomasses in energy generation, sugar cane. The management of this biomass can reduce costs compared to terrestrial biomasses, without the need for irrigation, fertilizers or other agricultural inputs. The ability to realize two daily collections proves its unique potential for biorefinery. On the positive side, it reinforces its biological characteristics with enhanced photosynthesis four times superior to terrestrial biomass, indeed, a high point to self-sufficient and sustainable biomass production systems with cycle carbon sequestration. Macroalgae pellets have a high calorific value of 20.19 MJ/Kg, with the advantage that they do not interfere with the supply of edible food and do not require large areas of land for cultivation, as is the case with terrestrial biomass. Positive results of marine macroalgae biomass calorific value can range new research and advanced technologies for the production of renewable energy, with reduced costs and environmental balance, promoting a progressive industrial scale in the production of energy with natural resources from the oceans.

## Figures and Tables

**Table 1:** Macroalgae species—Higher calorific value (HCV) and lower calorific value (LCV).

Species	Phylum	HCV/MJ/Kg	LCV/MJ/Kg
*Cryptonemia luxurians*	Rhodophyta	11.43	10.04
*Sargassum* sp.	Ochrophyta	10.68	9.67
*Ulva fasciata*	Chlorophyta	8.21	6.76
*Cryptonemia crenulata*	Rhodophyta	12.02	10.87
*Lobophora variegata*	Ochrophyta	10.58	9.58
*Gracilaria* sp.	Rhodophyta	11.14	9.91
*Cryptonemia seminervis*	Rhodophyta	9.67	8.99
*Ulva lactuca*	Chlorophyta	11.43	9.38
*Hydropuntia cornea*	Rhodophyta	11.42	9.86
*Padina* sp.	Ochrophyta	8.32	7.31
*Caulerpa microphysa*	Chlorophyta	6.37	5.97
*Hypnea pseudomusciformis*	Rhodophyta	8.73	6.51
*Sargassum vulgare*	Ochrophyta	11.19	9.79
Average	-	10.09	8.82

**Table 2:** Nutrient concentrations macroalgae species founding in the beaches of Maceió, Alagoas, Brazil.

Species/Biomass	Weight/g	Carbon %	Hydrogen %	Nitrogen %	Phosphorus g/Kg	Potassium g/Kg
*Cryptonemia* sp.	3.1	37.94	6.54	3.74	0.730	3.113
*Sargassum* sp.	3.1	28.99	4.78	1.30	0.048	19.869
*Ulva fasciata*	3.1	29.51	6.86	1.62	0.236	5.691
*Cryptonemia crenulata*	2.8	30.42	5.44	3.03	0.582	8.022
*Lobophora variegata*	3.1	30.62	4.72	1.19	0.470	7.606
*Gracilaria* sp.	2.9	33.62	5.8	2.55	0.225	34.872
*Cryptonemia seminervis*	3	20.60	3.22	2.35	1.255	6.197
*Ulva lactuca*	2.9	50.08	9.64	4.18	0.301	4.819
*Hydropuntia cornea*	3.1	41.97	7.33	0.95	ND	34.014
*Padina* sp.	3.1	28.21	4.79	1.59	0.284	9.181
*Caulerpa microphysa*	3	16.58	1.88	0.98	1.186	1.466
*Hypnea pseudomusciformis*	3	61.87	10.44	4.79	0.658	50.068
*Sargassum vulgare*	2.9	38.60	6.6	3.33	0.595	9.412
Aggregate biomass	2.9	43.97	6.73	4.53	0.593	8.795

ND—Not detected.

**Table 3:** Fibre components of 11 seaweeds species and aggregate biomass.

Seaweed Species	Phylum	Lignin (%)	Cellulose (%)	Ashes (%)
*Sargassum* sp.	Ochrophyta	9.41	11.76	8.06
*Cryptonemia crenulata*	Rhodophyta	2.44	9.27	2.46
*Lobophora variegata*	Ochrophyta	5.27	23.29	4.04
*Gracilaria* sp.	Rhodophyta	1.98	7.17	3.16
*Cryptonemia seminervis*	Rhodophyta	4.11	8.33	3.86
*Ulva lactuca*	Chlorophyta	9.13	9.57	4.15
*Hydropuntia cornea*	Rhodophyta	0.31	5.13	1.86
*Padina* sp.	Ochrophyta	12.72	10.42	10.56
*Caulerpa microphysa*	Chlorophyta	13.74	13.92	9.79
*Hypnea pseudomusciformis*	Rhodophyta	8.58	8.23	7.32
*Sargassum vulgare*	Ochrophyta	7.13	17.72	4.33
Aggregate biomass	-	7.29	12.01	3.75
Overall average		6.81	11.28	5.30

**Table 5:** Tide table on the days of macroalgae collections.

Date	Hours	Tide Height/m	Moon Phases	
12 March 2015	13.47 m	0.6	full moon	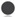
4 April 2015	9.47 m	0.2	full moon	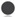
5 April 2015	10.15 m	0.2	full moon	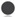
5 March 2016	7.11 m	0.6	waning moon	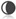
12 March 2016	12.09 m	0.1	new moon	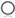
17 September 2016	10.00 m	0.0	full moon	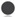
15 October 2016	8.56 m	0.0	waxing moon	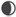
16 October 2016	9.38 m	0.0	full moon	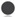

## Data Availability

The results of this research can be publicly consulted in: “Digital Repository Data of Pernambuco Federal University” at the link: https://repositorio.ufpe.br/handle/123456789/30792, accessed on 10 December 2024. All statistical and methodological data of research were actualized and confirmed with consent of all authors. The authors declare that they had support of Federal University of Pernambuco, Federal University of Alagoas and Northeast Regional Centre for Nuclear Sciences to carry out laboratory research and chemical analysis of the raw material.
